# Photobiomodulation on isolated mitochondria at 810 nm: first results on the efficiency of the energy conversion process

**DOI:** 10.1038/s41598-024-61740-w

**Published:** 2024-05-14

**Authors:** Andrea Amaroli, Mario Rene Clemente Vargas, Claudio Pasquale, Mirco Raffetto, Silvia Ravera

**Affiliations:** 1 Department of Earth, Environment and Life Sciences, University of Genoa, Corso Europa 26, 16132 Genoa, Italy; 2 Department of Electrical, Electronic, Telecommunications Engineering and Naval Architecture, University of Genoa, Via Opera Pia 11a, 16145 Genoa, Italy; 3 Department of Mechanical, Energy, Management and Transport Engineering, University of Genova, Via Opera Pia 15, 16145 Genoa, Italy; 4 Department of Experimental Medicine, University of Genoa, Via L. B. Alberti 2, 16132 Genoa, Italy

**Keywords:** Low-level light therapy, Photobiomodulation, Laser biostimulation, Phototherapy, Photostimulation, Near-infrared band, Electromagnetic field, Computer assisted dosimetry calculation, Mitochondria, Energy conversion, Efficiency, Thermodynamic, Cell biology, Medical research, Optics and photonics

## Abstract

In this paper the photobiomodulation on isolated mitochondria of bovine liver is studied as a thermodynamic process of conversion of energy. This analysis is conducted by considering a particular set-up for the photobiomodulation experiments of interest. It allows, in particular, the computation of the electromagnetic field and the related energetic quantities in the stimulated organelles. The measurements of the excess of biochemical power density produced by the illuminated mitochondria are performed at regular time intervals after the experiments. The calculations and the measurements finally allow us to obtain the first results on the efficiency of the process of conversion of electromagnetic energy into excess of biochemical energy released by the isolated organelles.

## Introduction

The term photobiomodulation (PBM) refers to the effects that can be induced on cells by incident electromagnetic waves in the optical or near-infrared bands^1^.

From a medical perspective, the interaction between light and non-plant cells offers the possibility of modulating pain, inflammation, and tissue healing and repair processes, just to name a few examples^1–4^.

It is now well established that one of the main actors in photobiomodulation processes is the mitochondrion^5^. Several photoacceptors, represented by cytochromes, are present within the mitochondria; they are able to respond with different affinities to visible and near-infrared wavelengths while capturing energy^5^. For instance, it has been described that flavins in Complex II can predominantly absorb blue light, while the cytochrome c oxidase in mitochondrial Complex IV is activated by visible light in the red and near-infrared spectrum (635, 810, 980, 1064 nm), along with Complex III responding to 810, 980, and 1064 nm, and Complex I to 1064 nm wavelengths^6–9^.

However, reducing photobiomodulation phenomena to a single light-mitochondria interaction may limit understanding in a complex environment such as a cell. For this reason, more recent research has highlighted possible interactions of light with other molecules, such as thiol-proteins, activated latent transforming growth factor-$\beta$1, water, and lipids^10–13^.

The description of the therapy also leads to confusion and debate. While for a drug, the predictability of therapeutic effects may be partially linked to the posology, in the case of photobiomodulation there is still no general agreement on the definition of the dose^14,15^. For example, the fluence is considered as a quantity of fundamental importance by some researchers but others express their concern about it^15^.

Overall, this unclear understanding of the phenomenon at the molecular, cellular, and tissue levels, along with the difficulty of standardizing the observed effects, has led to an incomplete acceptance of this therapy in many medical fields, despite a robust literature aimed at demonstrating the positive effects of photobiomodulation^16,17^.

In order to work on one of the established mechanisms of photobiomodulation, reducing the number of variables involved, the possibility of isolating mitochondria while preserving their enzymatic and energetic activities represents an excellent simplification to understand the mechanisms underlying light-cell interactions^7,18^. On the other hand, isolating from the cellular context should lead to caution in generalizing observed phenomena. However, in previous studies, we have highlighted the consistency of the observed data, first on extracted mitochondria^7,8^ and subsequently on cells^19,20^, tissues, and organisms^21–25^.

Recently, new interpretative approaches to photobiomodulation have been proposed, aiming to overcome the classical interpretation in terms of properties of the wave irradiated by the source, to analyze the phenomenon from the standpoint of the electromagnetic field capable of inducing biological reactions^26^ and to describe the entire process in terms of the thermodynamic principle of energy transfer^27^.

In this work we integrate the two approaches mentioned above. In particular, by using the experimental and modelling approach proposed in^26^, we have considered the possibility of calculating, with a controlled degree of uncertainty, the electromagnetic fields stimulating isolated mitochondria during PBM experiments. From the knowledge of the electromagnetic field, it is possible to easily deduce the active power and the total energy absorbed by mitochondria. In accordance with a procedure of irradiation and metabolic/energetic evaluation of isolated mitochondria, standardized in previous works^7,28^, the excess of biochemical power density produced by illuminated organelles compared to non-irradiated mitochondria will be measured. This phenomenon will be monitored immediately after the experiments and at regular time intervals. Thus, PBM will be characterized as a thermodynamic process of convertion of electromagnetic energy absorbed by mitochondria into excess of chemical energy produced by the same organelles, as suggested by Young and colleagues^27^, and as classically occurs, for example, in photosynthesis^29^. The values of efficiency of these energy conversion processes will be deduced and discussed.

Although the proposed evaluations can be done at different wavelengths, all results of this work are obtained at 810 nm.

## Materials and methods

### Mitochondria enriched-fraction isolation

In accordance with our previously established protocol^8^, bovine liver samples were procured from two female and two male cattle, all under one year of age, sourced from the Ceva slaughterhouse in Torino, Italy. The cattle were specifically bred for human consumption, adhering to the guidelines set by the Italian Ministry of Agricultural, Food, and Forestry Policies. The samples were collected and processed immediately after slaughter, adhering to all safety regulations. Importantly, as the animals were neither bred nor sacrificed at the University of Genoa, ethical committee approval was deemed unnecessary.

The isolation of the mitochondria-enriched fraction involved a series of steps. Initially, the bovine liver was washed in PBS and subsequently homogenized in a buffer composed of 0.25 M sucrose, 0.15 M KCl, 10 mM Tris-HCl (pH 7.4), and 1 mM EDTA. The resulting homogenate underwent centrifugation at 800 g for 10 minutes. The supernatant obtained was filtered and then subjected to centrifugation at 12,000 g for 15 minutes. The resulting pellet was resuspended in a different buffer consisting of 0.25 M sucrose, 75 mM mannitol, 10 mM Tris-HCl (pH 7.4), and 1 mM EDTA. The final supernatant from this step underwent centrifugation at 12,000 g for 15 minutes, and the mitochondrial pellet obtained was resuspended in the same buffer^7^.

### Devices and irradiation setup

**Figure 1:**
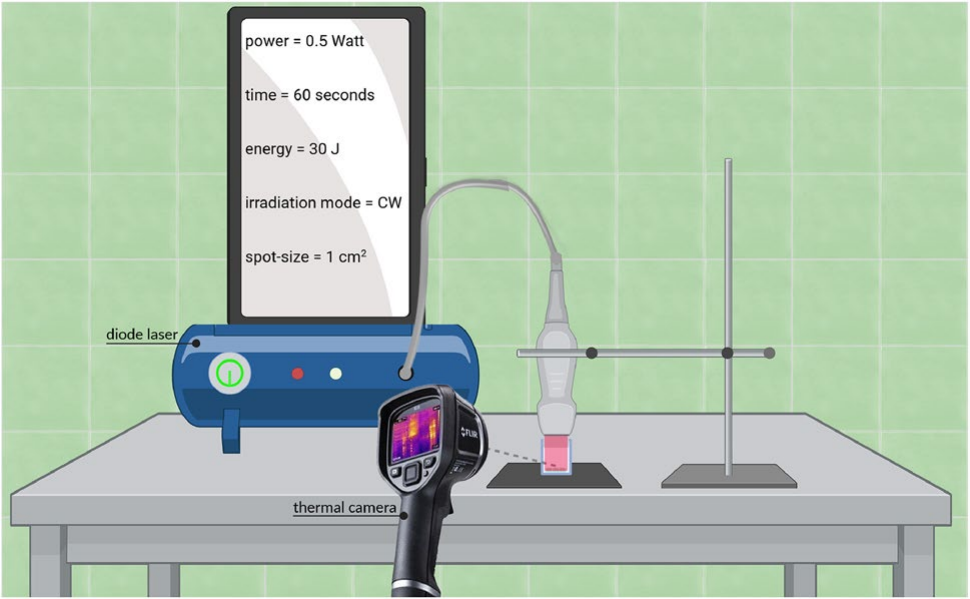
In our irradiation setup a laser equipped with a flat-profile hand-piece is used to illuminate isolated mitochondria in a cuvette placed on a light-absorbing sheet. A thermal camera is used to monitor the temperature of mitochondria during the experiments.

The devices and irradiation setup are shown in Fig. 1.

The irradiations were performed by using an ENEA 810-nm wavelength laser diode produced by Garda Laser (37024 Negrar, Verona, Italy). Most of its technical features can easily be found in the company website.

The laser was equipped with the hand-piece with a flat profile considered in^30^, which was fixed to a stand at a distance of 42 mm from the top of the workbench. It allows a uniform energy delivery on the irradiated area of 1 cm$^2$^30^. As per findings from our earlier investigations, the flat-top hand-piece demonstrates the ability to uniformly expose a 1 cm$^2$ circular spot area with a uniform energy distribution, which remains largely independent of the distance^30,31^.

A Metal Velvet light-absorbing panel, produced by Acktar Ltd. (8643 Kiryat-Gat, Israel), is laid on the workbench of our laboratory. The technical features of the absorbing sheet are described in the company website. In the presence of the sheet the reflection is almost negligible and this is useful to make the experiments independent of the characteristics of the workbench.

All irradiations were carried out with the cuvette placed on the light-absorbing panel. The cuvette is made of glass, has a cylindrical shape and a flat bottom. The thickness of the glass, the total height and the outer diameter of the chambers were 1.2 mm, 1.2 cm and 1.5 cm, respectively.

Isolated mitochondria of bovine liver were diluted in a saline solution containing 0.1 M Tris-HCl (pH 7.4), 0.1 M KCl, 5 mM MgCl$_2$, 0.2 mM P1,P5-Di(adenosine-5’) pentaphosphate, 0.6 mM ouabain, and 5 mM KH$_2$PO$_4$^26^. The resulting suspension always had a 300 $\mu$l volume, with just one part out of 64 of mitochondria (80 $\mu$g of total proteins).

Before irradiation, coherence between the power set on the device and the delivered power was evaluated using a power meter PM160T-HP (dynamic range: 0.01 W−70 W; ThorLabs, Bergkirchen, Germany).

The temperature of the mitochondria in the solution during delivery was monitored using a thermal camera, specifically the FLIR ONE Pro-iOS thermal camera (dynamic range: − 20 $^\text {o}$C / +400 $^\text {o}$C; resolution 0.1 $^\text {o}$C; FLIR Systems, Inc., designs, Portland, OR, USA).

### Irradiation parameters

Building upon a characterization performed in our previous work^7,32–34^, in this study, we first conducted a screening to identify experimental parameters capable of stimulating the mitochondria. Subsequently, we focused our attention on a representative value of fluence/energy. For this case we carried out a deeper analysis and deduced the first results on the efficiency of the process of conversion of electromagnetic energy into excess of biochemical energy released by the isolated organelles.

#### Screening experiments

A 635 nm light pointer of negligible power ($<0.5$ mW) was used to visualize the exposed circular area of 1 cm$^2$. After that, the samples were irradiated at 810 nm with 0.25, 0.5, 1 or 2 W for 30, 60 or 120 s. The corresponding values of fluence/energy or of energy density per unit area are reported in Table 1.

**Table 1:** Values of fluence/energy (expressed in J) or of energy density per unit area (expressed in J/cm$^2$) considered in our screening experiments.

		Irradiated power at 810 nm			
		0.25 W	0.5 W	1 W	2 W
Different durations of irradiation	30 s	7.5	15	30	60
	60 s	15	30	60	120
	120 s	30	60	120	240

The control was performed in the same way but, after a few seconds of light pointer irradiation, the device was set to irradiate 0 W for 30, 60 or 120 s (that is 0 J and 0 J/cm$^2$).

#### Irradiation for a representative value of fluence/energy

The cuvette position was determined by using the 635 nm light pointer, as in the previous cases. Then, the samples were irradiated at 810 nm with 0.25 W for 120 s, 0.5 W for 60 s, 1 W for 30 s and, finally, 2 W for 15 s. In all cases the fluence/energy is set to 30 J, corresponding to 30 J/cm$^2$.

The control was prepared in the same way as above.

### ATP synthesis evaluations

ATP synthesis was evaluated by using the highly sensitive luciferin/luciferase chemiluminescent method^24,23^. For any evaluation 10 $\mu$l of irradiated mitochondrial suspensions or controls were added to 40 $\mu$l of a saline solution composed of: 0.1 M Tris-HCl (pH 7.4), 0.1 M KCl, 5 mM MgCl$_2$, 0.2 mM P1,P5-Di(adenosine-5’) pentaphosphate, 0.6 mM ouabain, 5 mM KH$_2$PO$_4$, with 5 mM pyruvate and 5 mM malate. Afterwards, 50 $\mu$l of luciferin/luciferase solution was added, and the addition of 0.1 mM ADP started the reaction.

All measurements were performed by using a Glomax 20/20 luminometer manufactured by Promega Corporation (Madison, Wisconsin, USA).

#### Evaluations after screening experiments

The ATP synthesis assays were performed immediately after the irradiations and monitored for 2 minutes. The measurements were repeated 10 and 15 minutes after the irradiations.

#### Evaluations for a representative value of fluence/energy

The ATP synthesis evaluations were performed immediately after the irradiations and, as before, they were monitored for 2 minutes. The same operation was repeated after 2 or 5 minutes to evaluate the permanence of the post-irradiation effect. It was not necessary to do the same after a longer time because in all cases during the screening phase we verified that no post-irradition effect was present after 10 or 15 minutes.

### Statistical analysis

Our results are representative of at least 3 independent experiments. All data were analyzed with GraphPad Prism 8.0 software (GraphPad Software, San Diego, CA, USA). ATP synthesis was expressed as the mean value plus or minus the standard deviation and then analyzed using one-way ANOVA followed by Tukey’s multiple comparison test. Differences were considered statistically significant if the error probability was $p<0.05$.

### Models for the evaluation of electromagnetic dosimetry

**Figure 2:**
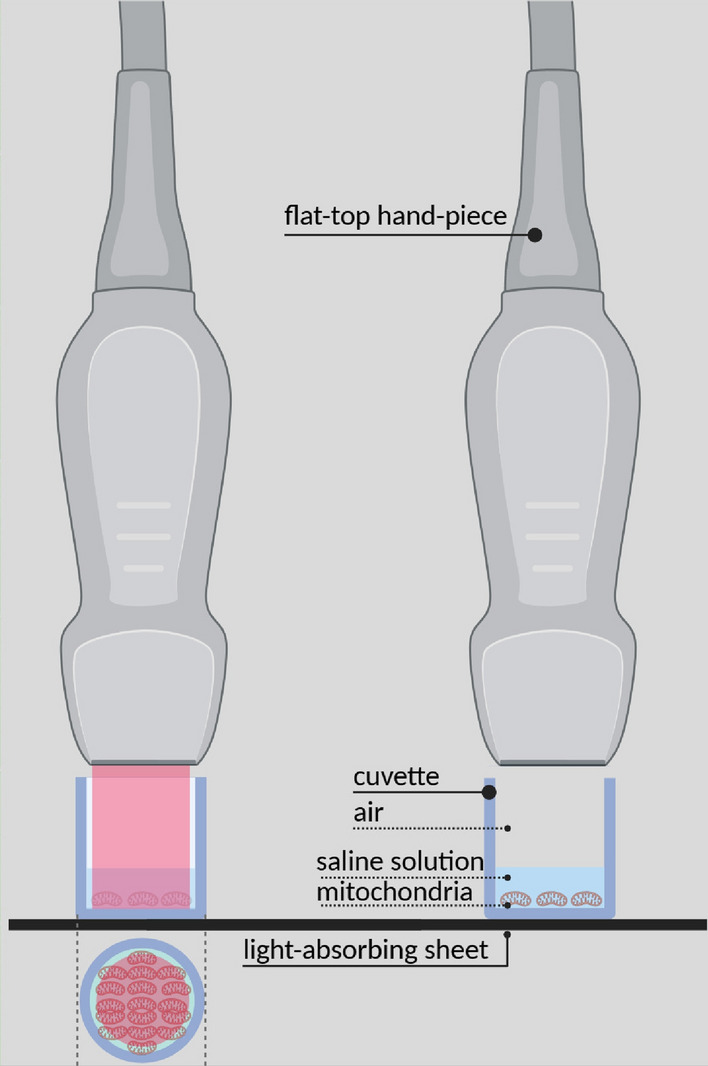
A closer view of the flat-top hand-piece, the irradiated beam and the mitochondria in the incubation chamber. The light of the pointer is visible on the left while the irradiating beam is not visible on the right. The mitochondria on the flat bottom of the cuvette are out of scale.

**Figure 3:**
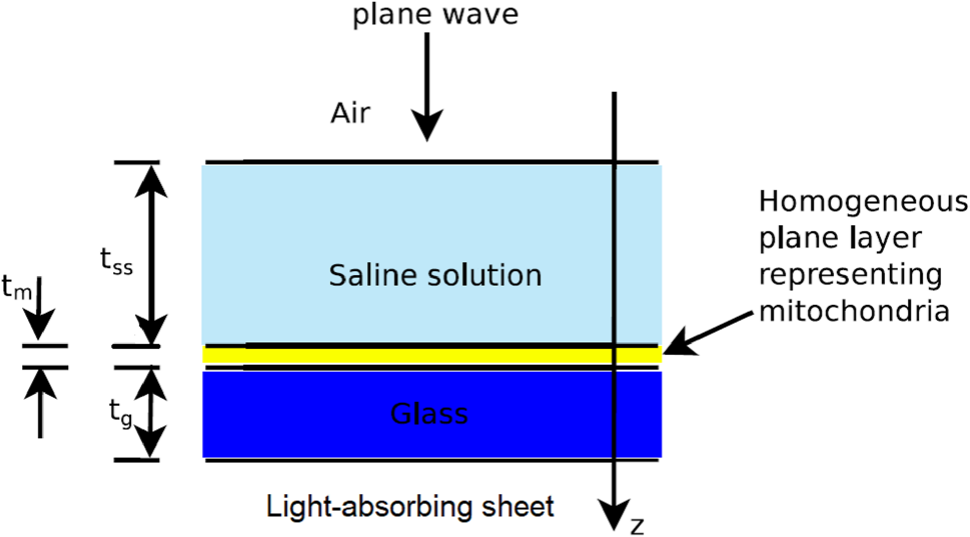
The one-dimensional model considered to approximate the electromagnetic quantities of interest.

The computation of the electromagnetic field inside mitochondria is not an easy task. Moreover, it can easily become an insurmountable problem if the illumination conditions and the geometry of the incubation chambers are not properly defined^26^. Our experimental set-up was defined to overcome this problem and ease the computations of interest^26^. In particular, as shown in Fig. 2, we used a flat-top hand-piece with a beam area of 1 cm$^2$, together with incubation chambers having internal areas of 1.247 cm$^2$. In this way we were able to avoid strong scattering effects from the vertical walls of the cuvettes^26^. Moreover, the mitochondria spread over the flat bottom of the cuvette and form a rather uniform and approximately plane layer. Finally, inside the beam the field amplitude is rather uniform^30^ and can be modelled as a plane wave^26^. For all these reasons, the electromagnetic field which stimulates more than 80% of the mitochondria involved in photobiomodulation experiments could be calculated by using a rather simple one-dimensional model, such as that shown in Fig. 3.

The one-dimensional model of interest is made up of five layers. The first four of these layers, starting from the side of the source, are made up of air, saline solution, mitochondria and glass. They are the same as in the model shown in Fig. 2 of^26^. The fifth layer, instead, represents the light-absorbing sheet which is present in our experimental set-up (see Fig. 1).

Models like that of Fig. 3 allow the computation of the electromagnetic field everywhere and, in particular, in the layer of mitochondria, in a tiny fraction of a second. This is an important feature for our study. As a matter of fact, different PBM experiments can involve distinct incubation chambers, slightly different quantities of mitochondria and non-identical volumes of saline solution, just to cite a few dissimilarities. In order to understand the effects of all these changes we need to solve different problems by considering different parameters in our model. Moreover, the effective constitutive parameters of mitochondria are known only approximatively, at the wavelength of interest. Thus, in order to estimate the electromagnetic field of interest other parameters of our model need to be changed.

In order to take account of all these considerations, a what-if analysis is unavoidable and a very large number of simulations are required.

## Results

### Results of screening experiments

**Figure 4:**
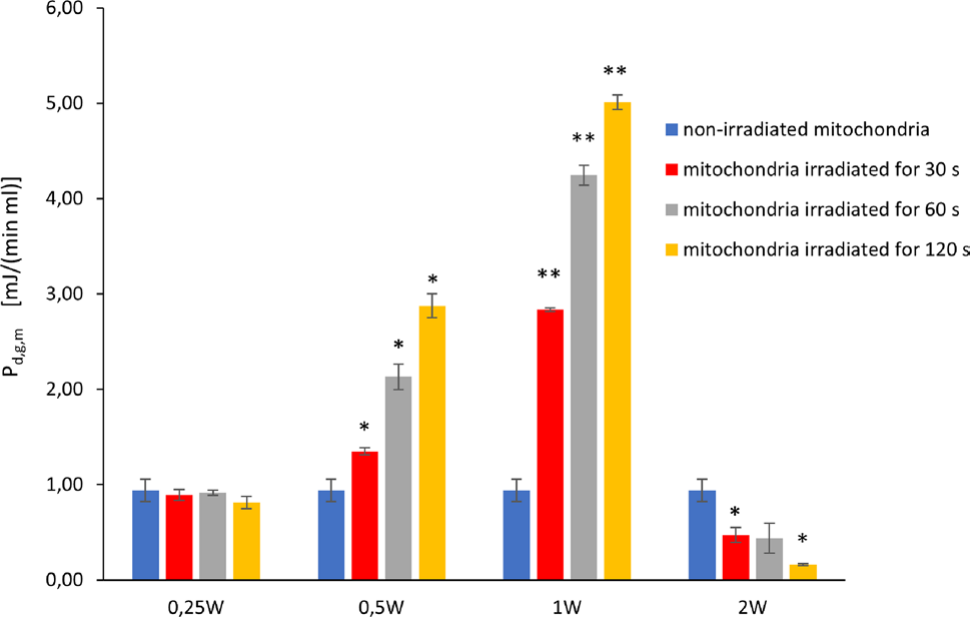
Behaviour of $P_{d,g,m}$, the biochemical power density produced by mitochondria, under different irradiation conditions. The height of any bar indicates the mean value of the quantity of interest. The mean is calculated by using the results of three independent experiments, for any of the considered cases. Any bar has a black range at the top: it provides information on the value of the standard deviation; the range is given by the mean value to which the standard deviation is added or subtracted. Four groups of bars are shown: those on the left correspond to a laser output power of 0.25 W; the second group from the left is obtained by setting the same parameter to 0.5 W; further on the right, the results are those obtained by using 1.0 W; the last group on the right corresponds to the case of 2 W. The blue bar in any group provides the mean value of the quantity of interest for non-irradiated mitochondria. The information is repeated to ease data comparison. The other colors of the bars indicate different durations of irradiation: red for 30 s, gray for 60 s and yellow for 120 s. *, ** indicate a significant difference for $p<0.05$ and 0.01, respectively, between one time and the next within the same laser treatment.

The results obtained from the screening experiments are reported in Fig. 4. In particular, the figure shows the behaviour of $P_{d,g,m}$, the biochemical power density produced by mitochondria, under different irradiation conditions. All values of $P_{d,g,m}$ were easily deduced from the results of ATP synthesis by a trivial convertion of quantities (since a nmol/(min $\mu$l) of ATP correspond to 30.5 mJ/(min ml) $\simeq 0.508$ mW/ml). The indicated results of ATP synthesis were instead achieved immediately after the irradiation, as described in Sect. "ATP synthesis evaluations".

Any bar in the figure represents a mean value. At the top of a bar one can find a range, given by the mean value plus or minus the standard deviation.

Any group of bars corresponds to a given value of output power of the laser: 0.25 W for the group on the left, 2 W for that on the right. In the middle one finds the groups obtained with 0.5 W and 1 W.

The blue bar in any group provides the mean value of the quantity of interest for non-irradiated mitochondria. The information is repeated in all groups to ease data comparison. The other colors of the bars indicate different durations of irradiation: red for 30 s, gray for 60 s and yellow for 120 s.

The figure shows that there is no significant effect when the output power of the laser is set to 0.25 W, independently of the duration of irradiation. When the power is equal to 2 W the mitochondria appear to be slightly inhibited. On the contrary, one can observe significant stimulating effects on mitochondria when the laser output power is 0.5 W or 1 W. In particular, these effects becomes larger and larger as the duration of the experiment increases.

As it was pointed out in Sect. "Irradiation for a representative value of fluence/energy", we obtained no post-irradiation effect 10 or 15 minutes after irradiation. For the indicated reason, no data are provided about these additional assessments.

Evaluations of irradiated power values using a power meter highlighted the consistency of the experimental setup. Moreover, the absence of significant temperature variations during the experiments was verified by the thermal camera described in Sect. "Devices and irradiation setup".

### Results for a representative value of fluence/energy

**Figure 5:**
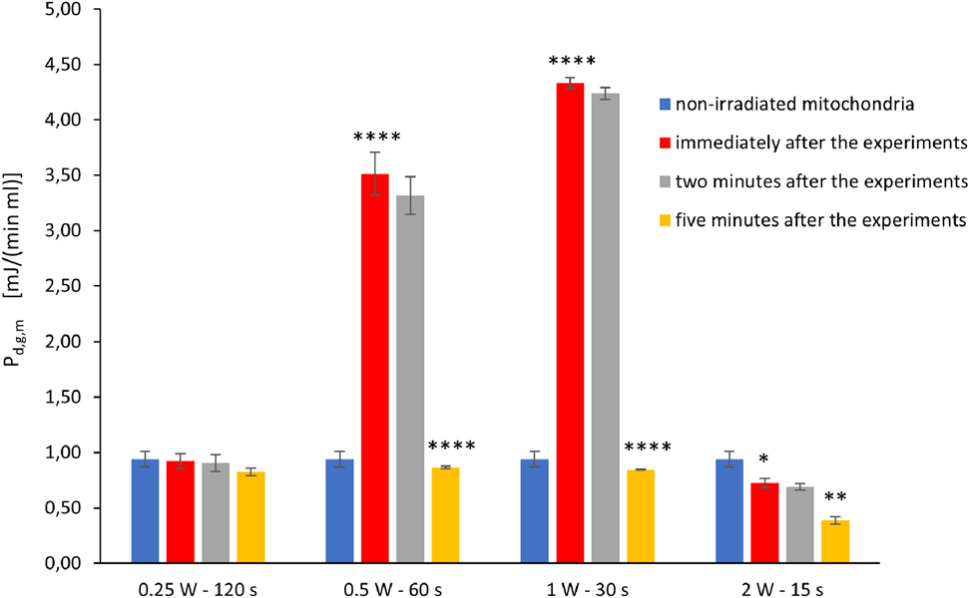
Behaviour of $P_{d,g,m}$, the biochemical power density produced by mitochondria, under different irradiation conditions. The height of any bar indicates the mean value of the quantity of interest. The mean is calculated by using the results of three independent experiments, for any of the considered cases. Any bar has a black range at the top: it provides information on the value of the standard deviation; the range is given by the mean value to which the standard deviation is added or subtracted. Four groups of bars are shown: they correspond to different values of laser output power and different durations of the experiments. From left to right one finds groups obtained for, respectively, 0.25 W and 120 s, 0.5 W and 60 s, 1.0 W and 30 s and 2 W and 15 s. The blue bar in any group always provides the same information: the mean value of the quantity of interest for non-irradiated mitochondria. The information is repeated to ease data comparison. The other colors of the bars indicate when the biochemical power density is measured. In particular, the data collected by measurements performed immediately after the irradiation are shown by red bars. Gray or yellow bars, instead, represent data measured two or five minutes after the end of the irradiation of mitochondria, respectively. *, **, **** indicate a significant difference for $p<0.05$, 0.01 and 0.0001, respectively, between one time after laser exposure and the next within the same laser treatment.

Figure 5 shows the values of $P_{d,g,m}$ obtained by setting the fluence/energy to 30 J, as described in Sect. "Irradiation for a representative value of fluence/energy".

In these cases, too, a bar represents a mean value with an indication of the magnitude of the standard deviation. As before, the blue bars represent the biochemical power density produced by non-irradiated mitochondria. However, the other colors of the bars indicate when the biochemical power density was measured, according to the indications reported in Sect. "Evaluations for a representative value of fluence/energy": red bars for measurements performed immediately after the irradiation; gray or yellow bars, instead, represent data measured two or five minutes after the end of the irradiation of mitochondria, respectively.

As expected from the results of Fig. 4, mitochondria are not stimulated in the two cases 0.15 W–120 s or 2 W–15 s. The differences with respect to the corresponding data obtained with non-irradiated mitochondria are not meaningful, in practice.

However, when the laser output power was set to 0.5 W for 60 s the values of $P_{d,g,m}$ were much larger than those obtained for non-irradiated organelles, and this happened in particular immediately after the experiment and two minutes later. However, the triggering effects were completely lost after five minutes.

From a qualitative point of view we got the same results from the experiments carried out by setting the laser output power to 1.0 W for 30 s. Immediately after the laser switch-off we got a mean value of $P_{d,g,m}$ of 4.334 mJ/(min ml). The mean slightly decreased to 4.237 mJ/(min ml) after two minutes and then it dropped to $8.457 \, 10^{-1}$ mJ/(min ml) after five minutes.

### Results of the numerical what-if analysis

Our what-if analysis is based on 37800 simulations. The constitutive parameters of the saline solution and of the glass of which the incubation chambers are made of are known with great precision at the wavelength of interest^35–37^. Thus, there is no need to consider different values for these parameters. In particular, we set the refractive index of the saline solution, $n_{ss}$, to $1.331-j1.257 \ 10^{-7}$. The same parameter for the glass layer is $n_g=1.511-j1.096 \ 10^{-8}$.

All other parameters of our model, instead, assume different values. In particular, the light-absorbing sheet is characterized by the producer by a low reflectance, smaller than or equal to 0.01 in the near infrared band. It corresponds to a magnitude of $\Gamma$, the reflection coefficient at any interface with air, smaller than or equal to 0.1. The constitutive parameters of the sheet are not known and to take account of it we consider a homogeneous medium characterized by the seven values of refractive index, $n_{las}$, reported in Table 2. The first three values on the left of Table 2 are chosen to get $|\Gamma | \simeq 0.1$ with a different balance of real and imaginary parts. The second group of three values from the left of the same table is defined in the same way to get $|\Gamma | \simeq 0.05$. The last value on the right, instead, obviously gives $|\Gamma | = 0$.

**Table 2:** Values of the refractive index of the homogeneous medium we considered to take account of the possible different features of the light-absorbing sheet.

1.222	1.196-j0.1	1-j0.201	1.111	1.093-j0.05	1-j0.1	1

The refractive index of the layer of mitochondria at 810 nm, $n_m$, must also assume different values in our what-if analysis. As a matter of fact, one can easily find different complex values for such a quantity in the open literature^38–40^. In particular, $n_m=n_m'-jn_m''$ can be assumed to have a real part $n_m'$ in the range [1.35, 1.45] and an imaginary part $n_m''$ belonging to $[1.289 \, 10^{-6},9.669 \, 10^{-6}]$^26^. In our analysis we take account of these uncertainties by considering 25 different complex values for $n_m$. They can be obtained by considering the possible combinations of the following sets of values: $n_m' \in \{ 1.35, 1.375, 1.40, 1.425, 1.45 \}$ and $n_m'' \in \{ 1.289 \ 10^{-6}, 3.384 \ 10^{-6}, 5.479 \ 10^{-6}, 7.574 \ 10^{-6}, 9.669 \ 10^{-6} \}$.

Finally, the nominal values of the thicknesses of the layers of saline solution, $t_{ss,nv}$, mitochondria, $t_{m,nv}$, and glass, $t_{g,nv}$, are equal to 2.368 mm, 37.59 $\mu$m and 1.2 mm, respectively. The last of these values is indicated by the producer of the incubation chambers. As for the first two, instead, they are determined by considering the nominal value of the internal area of the incubation chambers, equal to 124.7 mm$^2$, and volumes of $\frac{63}{64} \ 300$ mm$^3$ and $\frac{1}{64} \ 300$ mm$^3$, respectively. As noted before, they could change from one experiment to another. For example, the external diameter and thickness of the incubation chambers are known to have uncertainties equal to $\pm 0.2$ mm and $\pm 0.05$ mm, respectively. For this reason, we need to consider a set of values for these quantities. In all considered layers we have a standing wave due to the superposition of progressive and regressive waves in each medium^41^ (pp. 110-114),^42^ (pp. 98-101). They are attenuating standing waves because all media present losses. However, the standing wave patterns for the electric and magnetic fields are affected by such losses in a marginal way in our experiments. This can be easily understood by calculating the skin depths for the media of interest, at 810 nm. With the indicated parameters one easily finds that they are equal to 1.026 m for the saline solution and 11.76 m for glass. Moreover, even considering the largest losses among those considered above, one gets a skin depth for mitochondria equal to 1.333 cm. Since in all cases these values are much larger than the nominal thicknesses of the corresponding layers, for these considerations we can neglect attenuation and consider the standing waves as periodic in the considered layers. Their period is known^43^ (pp. 92-93) to be equal to half the wavelength in the considered media, $\lambda _{ss}$, $\lambda _m$, $\lambda _g$, which are equal to 0.6085 $\mu$m, 0.5786 $\mu$m and 0.5361 $\mu$m, respectively. Our what-if analysis can then be limited to consider variations of the thickness of a given layer by adding or subtracting a quarter wavelength to its nominal value. In particular, if the nominal value of the thickness of the layer of interest is denoted by $t_{li,nv}$ and the corresponding wavelength by $\lambda _{li}$, by exploiting the indicated periodicity, we can limit the thickness of that layer to assume one of the following six values: $t_{li} \in \{ t_{li,nv} - \frac{\lambda _{li}}{4}, t_{li,nv} - \frac{\lambda _{li}}{6}, t_{li,nv} - \frac{\lambda _{li}}{12}, t_{li,nv}, t_{li,nv} + \frac{\lambda _{li}}{12}, t_{li,nv} + \frac{\lambda _{li}}{6} \}$. In practice, we consider the 216 possible different combinations which can be obtained by picking for $t_{ss}$, $t_m$ and $t_g$ one of the values reported in Table 3.

**Table 3:** Values of the ticknesses of the layers of saline solution, mitochondria and glass, expressed in nm.

	Possible thickness values of the layers					
	$t_{li,nv} - \frac{\lambda _{li}}{4}$	$t_{li,nv} - \frac{\lambda _{li}}{6}$	$t_{li,nv} - \frac{\lambda _{li}}{12}$	$t_{li,nv}$	$t_{li,nv} + \frac{\lambda _{li}}{12}$	$t_{li,nv} + \frac{\lambda _{li}}{6}$
Saline solution mitochondria glass	2367847.9	2367898.6	2367949.3	2368000	2368050.7	2368101.4
	37445	37494	37542	37590	37638	37686
	1199865.9	1199910.6	1199955.3	1200000	1200044.7	1200089.4

All 216 possible combinations of values are considered in our what-if analysis. All thicknesses can be calculated as indicated in the second row of the table by considering $t_{ss,nv}=2.368 \ 10^6$ nm and $\lambda _{ss}=608.5$ nm for the layer of saline solution, $t_{m,nv}=37.59 \ 10^3$ nm and $\lambda _m=578.6$ nm for the layer of mitochondria and $t_{g,nv}=1.2 \ 10^6$ nm and $\lambda _g=536.1$ nm for the layer of glass.

In all simulations we consider an incident plane wave with a power density per unit area of 1 W/cm$^2$. Considering that just one square centimeter of mitochondria is actually illuminated, the simulations directly provide results for $P_{a,m}$, the average (in time) electromagnetic power absorbed by mitochondria^26^. These results are clustered into five well-defined groups. Each group corresponds to a specific value of $n_m''$. For all values of $n_m''$ considered in our analysis, Table 4 reports the average values $\overline{P_{a,m}}$ of the corresponding 7560 results of $P_{a,m}$, together with the corresponding standard deviation $\sigma (P_{a,m})$. Both quantities are expressed in mW. From these data one easily deduces that the other changing parameters of our what-if analysis have much smaller effects on the results of $P_{a,m}$ than $n_m''$.

**Table 4:** Average value, $\overline{P_{a,m}}$, and standard deviation, $\sigma (P_{a,m})$, for the different groups of results of $P_{a,m}$. Each group, which is made up of 7560 numbers, corresponds to a specific value of $n_m''$.

	$\overline{P_{a,m}}$	$\sigma (P_{a,m})$
$n_m''=1.289 \ 10^{-6}$	0.7551	0.03026
$n_m''=3.384 \ 10^{-6}$	1.981	0.07930
$n_m''=5.479 \ 10^{-6}$	3.205	0.1282
$n_m''=7.574 \ 10^{-6}$	4.428	0.1768
$n_m''=9.669 \ 10^{-6}$	5.649	0.2253

These values are reported on the left column of the table.

Overall, $P_{a,m}$ is contained in the range [0.6985, 6.381] mW. The global average value and standard deviation are equal to 3.204 and 1.737, respectively. By the linearity of the model, one can immediately deduce the corresponding ranges for the other illuminating fields considered in our experiments: for a power density per unit area of *k* W/cm$^2$ the range becomes $[0.6985 \, k, 6.381 \, k]$ mW. Finally, due to the different exposure times, the energy absorbed by the organelles during the experiments, $E_{a,m}$, was in any case in the range [20.95, 191.4] mJ. These numbers are deduced, for example, by multiplying the minimum and maximum values of $P_{a,m}$ indicated above, for the case $k=1$, by 30 s. In order to estimate the range of values of the energy density absorbed by mitochondria, $E_{d,a,m}$, we take into account that the organelles can spread in an incubation chamber of 1.247 cm$^2$ and that just 1 cm$^2$ is actually illuminated. Then, $E_{d,a,m} \ge \frac{20.95}{4.6875} \cdot 1.247=5.573$ $mJ/\mu$l and $E_{d,a,m} \le \frac{191.4}{4.6875} \cdot 1.247=50.91$ $mJ/\mu$l.

### Results about the excess of biochemical energy density produced by illuminated mitochondria

From the results for a representative value of fluence/energy one can easily deduce the excess of biochemical power density produced by illuminated organelles with respect to that obtained by non-irradiated mitochondria. As already explained, this can be done immediately after the experiment or after two or five minutes. We will denote these quantities by $\Delta P_{d,g,m}(t=0)$, $\Delta P_{d,g,m}(t=120)$ and $\Delta P_{d,g,m}(t=300)$, respectively.

**Figure 6:**
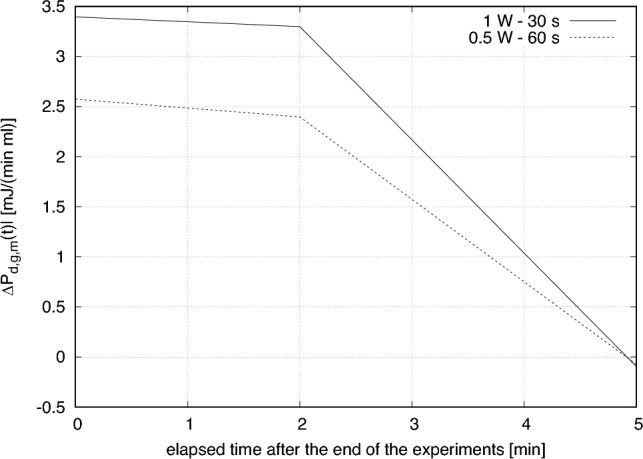
Behaviour of $\Delta P_{d,g,m}(t)$, the excess of biochemical power density produced by illuminated organelles with respect to that obtained by non-irradiated mitochondria, as a function of the elapsed time after the end of the experiments. The plots refer to two cases: when the laser output power is equal to 0.5 W and the exposure time is 60 s or when the output power is increased to 1 W and the exposure time is reduced to 30 s.

Since we observed no significant effect for the two cases characterized by 0.25 W - 120 s and by 2 W - 15 s, in the following we consider only the data for the other two cases. In particular, for the case 0.5 W - 60 s we get $\Delta P_{d,g,m}(t=0)=2.574$ mJ/(min ml), $\Delta P_{d,g,m}(t=120)=2.379$ mJ/(min ml) and $\Delta P_{d,g,m}(t=300)=-7.330 \ 10^{-2}$ mJ/(min ml), while for 1.0 W - 30 s we obtain $\Delta P_{d,g,m}(t=0)=3.395$ mJ/(min ml), $\Delta P_{d,g,m}(t=120)=3.298$ mJ/(min ml) and $\Delta P_{d,g,m}(t=300)=-9.340 \ 10^{-2}$ mJ/(min ml). The behaviours of $\Delta P_{d,g,m}(t)$ as functions of the elapsed time after the end of the experiments, for the two cases of interest, are reported in Fig. 6.

By integrating $\Delta P_{d,g,m}(t)$ with respect to time we get $\Delta E_{d,g,m}$, the excess of biochemical energy density produced by illuminated mitochondria during the five-minute period which follows the end of each experiment. With our sampled data, by assuming a linear interpolation, we have:

1 $$\begin{aligned} \Delta E_{d,g,m}= & {} \frac{\Delta P_{d,g,m}(t=0)+\Delta P_{d,g,m}(t=120)}{2} \, 2 + \nonumber \\{} & {} + \frac{\Delta P_{d,g,m}(t=120)+\Delta P_{d,g,m}(t=300)}{2} \, 3 \quad \text {[mJ/ml]}. \end{aligned}$$

In particular, we get $\Delta E_{d,g,m}=8.412$ mJ/ml when the laser power output is equal to 0.5 W and the exposure time is 60 s. When the power is doubled and the exposure time is halved we get $\Delta E_{d,g,m}=11.50$ mJ/ml.

## Discussion

As noted in the Introduction, in this section we try to characterize the photobiomodulation on isolated mitochondria from a thermodynamic point of view^27^, as an energy transfer process. It is a characterization of great interest, due to the specific functions performed by mitochondria inside cells^44,45^.

By using the results of Sects. "Results of the numerical what-if analysis" and "Results about the excess of biochemical energy density produced by illuminated mitochondria" we have that the energy absorbed by any milliliter of isolated mitochondria is in the range $E_{d,a,m} \in [5573, 50910]$ mJ/ml, while the excess of biochemical energy produced by a milliliter of illuminated mitochondria is $\Delta E_{d,g,m}=8.412$ mJ/ml, when the laser output power is equal to 0.5 W and the exposure time is 60 s, and $\Delta E_{d,g,m}=11.50$ mJ/ml, when the power is doubled and the exposure time is halved. We previously observed that the photobiomodulation on isolated mitochondria had no effects in the other cases.

For the two cases with significant photobiomodulation effects, the efficiency of the energy conversion process, $\eta$, can then be estimated to be larger than $\frac{8.412}{50910}=1.652 \ 10^{-4}$ and smaller than $\frac{8.412}{5573}=1.509 \ 10^{-3}$, for the former of the two cases of interest. The corresponding range for the other case is $[2.259 \ 10^{-4},2.064 \ 10^{-3}]$.

The results obtained for $\eta$ present significant uncertainties and should only be used to infer indications on the order of magnitude of such a quantity.

By far the most important errors in the above estimates are those related to the uncertainty with which the value of $n_m''$ is known. The next most important cause of error is due to the significant difference between the responses obtained from different biological materials subjected to the same treatment. For example, one can easily verify that for the case 0.5 W - 60 s the value of $P_{d,g,m}$ was equal to 2.13 mJ/(min ml) immediately after the screening experiments (see the gray bar in the second group of bars from the left in Fig. 4) and to 3.51 mJ/(min ml) immediately after the more detailed analysis for a representative value of fluence/energy (see the red bar in the second group of bars from the left in Fig. 5).

The other numerous causes of errors and uncertainties have a minor impact on the results and, as indicated above, the entire set-up was carefully designed with this in mind.

In spite of the indicated limitations, to the best of authors’ knowledge, the above are the first results about the efficiency of the energy transformation which takes place in PBM experiments on isolated mitochondria. It is interesting to observe that, when the photochemical reaction takes place, the efficiency is by no means negligible: it was estimated to be larger than $0.0165 \%$ with a possible largest value of $0.2 \%$. To better appreciate this conclusion, one can observe that green plants, which are specialized for light energy conversion, guarantee a maximum overall energy conversion of approximately $10 \%$ and that photosynthetic efficiency in actual “net field production” seldom exceeds $2 \%$, with typical values being in the neighborhood of $0.5 - 1.5 \%$^29^.

Before concluding this section, it could be important to note that the results shown in Fig. 5 are at odds with the principle of irradiance reciprocity: in our experiments the biological process triggered in isolated mitochondria by electromagnetic fields at 810 nm is not determined by the fluence alone and, as a consequence, the Roscoe-Bunsen law of reciprocity does not hold true. In particular, our results confirm the presence of limitations to the applicability of the principle, as it was highlighted for example in^46^ and^14^.

## Conclusions

A particular set-up was considered for the photobiomodulation on isolated mitochondria of bovine liver. It allows us to estimate the electromagnetic field and the related energetic quantities in photostimulated organelles. From these estimates and the results of the experiments a characterization of photobiomodulation as a thermodynamic process of energy conversion is achieved. In particular, for the first time to the best of authors’ knowledge, some results on the efficiency of the process are deduced. It is shown that, under specific conditions, the efficiency is by no means negligible.

The efficiency of PBM can be deduced at different wavelengths. However, all results discussed in this paper were obtained at 810 nm.

## Data Availability

The datasets used and/or analyzed during the current study are available from the corresponding author on reasonable request.
