# Author Correction: Photobiomodulation on isolated mitochondria at 810 nm: first results on the efficiency of the energy conversion process

**DOI:** 10.1038/s41598-024-72599-2

**Published:** 2024-09-13

**Authors:** Andrea Amaroli, Mario Rene Clemente Vargas, Claudio Pasquale, Mirco Raffetto, Silvia Ravera

**Affiliations:** 1 Department of Earth, Environment and Life Sciences, University of Genoa, Corso Europa 26, 16132 Genoa, Italy; 2 Department of Electrical, Electronic, Telecommunications Engineering and Naval Architecture, University of Genoa, Via Opera Pia 11a, 16145 Genoa, Italy; 3 Department of Mechanical, Energy, Management and Transport Engineering, University of Genova, Via Opera Pia 15, 16145 Genoa, Italy; 4 Department of Experimental Medicine, University of Genoa, Via L. B. Alberti 2, 16132 Genoa, Italy

Correction to: *Scientific Reports* 10.1038/s41598-024-61740-w, published online 14 May 2024

The original version of this Article contained errors in Table 4, where the last two values in column 1 were incorrect. The correct and incorrect values appear below.

Incorrect:

**Table:** 

$$\mathop{n}\nolimits_{m}^{\prime\prime } = 5.479 \, {10^{ - 6}}$$	
$$\mathop{n}\nolimits_{m}^{\prime\prime } = 5.479 \, {10^{ - 6}}$$	

Correct:

**Table:** 

$$\mathop{n}\nolimits_{m}^{\prime\prime } = 7.574 \, {10^{ - 6}}$$	
$$\mathop{n}\nolimits_{m}^{\prime\prime } = 9.669 \, {10^{ - 6}}$$	

The original Article has been corrected.

